# Selective inhibition of RNA polymerase I transcription as a potential approach to treat African trypanosomiasis

**DOI:** 10.1371/journal.pntd.0005432

**Published:** 2017-03-06

**Authors:** Louise E. Kerry, Elaine E. Pegg, Donald P. Cameron, James Budzak, Gretchen Poortinga, Katherine M. Hannan, Ross D. Hannan, Gloria Rudenko

**Affiliations:** 1 Department of Life Sciences, Sir Alexander Fleming Building, Imperial College London, London, United Kingdom; 2 ACRF Department of Cancer Biology and Therapeutics, The John Curtin School of Medical Research, The Australian National University, Canberra, Australia; 3 Oncogenic Signalling and Growth Control Program, Peter MacCallum Cancer Centre, Melbourne, Australia; Hunter College, CUNY, UNITED STATES

## Abstract

*Trypanosoma brucei* relies on an essential Variant Surface Glycoprotein (VSG) coat for survival in the mammalian bloodstream. High *VSG* expression within an expression site body (ESB) is mediated by RNA polymerase I (Pol I), which in other eukaryotes exclusively transcribes ribosomal RNA genes (rDNA). As *T*. *brucei* is reliant on Pol I for VSG transcription, we investigated Pol I transcription inhibitors for selective anti-trypanosomal activity. The Pol I inhibitors quarfloxin (CX-3543), CX-5461, and BMH-21 are currently under investigation for treating cancer, as rapidly dividing cancer cells are particularly dependent on high levels of Pol I transcription compared with nontransformed cells. In *T*. *brucei* all three Pol I inhibitors have IC50 concentrations for cell proliferation in the nanomolar range: quarfloxin (155 nM), CX-5461 (279 nM) or BMH-21 (134 nM) compared with IC50 concentrations in the MCF10A human breast epithelial cell line (4.44 μM, 6.89 μM or 460 nM, respectively). *T*. *brucei* was therefore 29-fold more sensitive to quarfloxin, 25-fold more sensitive to CX-5461 and 3.4-fold more sensitive to BMH-21. Cell death in *T*. *brucei* was due to rapid inhibition of Pol I transcription, as within 15 minutes treatment with the inhibitors rRNA precursor transcript was reduced 97-98% and *VSG* precursor transcript 91-94%. Incubation with Pol I transcription inhibitors also resulted in disintegration of the ESB as well as the nucleolus subnuclear structures, within one hour. Rapid ESB loss following the block in Pol I transcription argues that the ESB is a Pol I transcription nucleated structure, similar to the nucleolus. In addition to providing insight into Pol I transcription and ES control, Pol I transcription inhibitors potentially also provide new approaches to treat trypanosomiasis.

## Author summary

*Trypanosoma brucei* is protected by an essential Variant Surface Glycoprotein (VSG) coat in the mammalian bloodstream. The active *VSG* gene is transcribed by RNA polymerase I (Pol I), which typically only transcribes rDNA. Pol I transcription inhibitors are under clinical trials for cancer chemotherapy. As *T*. *brucei* relies on Pol I for VSG transcription, we investigated its susceptibility to these drugs. We show that quarfloxin (CX-3543), CX-5461, and BMH-21 are effective against *T*. *brucei* at nanomolar concentrations. *T*. *brucei* death was due to rapid and specific inhibition of Pol I transcription. Incubation with Pol I transcription inhibitors also resulted in disappearance of Pol I subnuclear structures like the nucleolus and the *VSG* expression site body (ESB). Rapid ESB loss followed the Pol I transcription block, arguing that the ESB is nucleated by Pol I transcription. Pol I transcription inhibitors could therefore potentially function as novel drugs against trypanosomiasis.

## Introduction

Human African Trypanosomiasis (HAT) or African Sleeping Sickness is endemic to sub-Saharan Africa, with distribution restricted by the tsetse fly insect vector [1]. Most of the HAT disease burden (98%) is the chronic form of the disease caused by *Trypanosoma brucei gambiense*. The remainder of the HAT cases are the acute form of the disease caused by *Trypanosoma brucei rhodesiense* [2]. Although there has been a progressive decline in the annual number of HAT cases, 1.8 million Africans are still thought to be living in high or very high risk areas, with 11.3 million people at moderate risk of contracting HAT [3]. Vaccines are ineffective against *T*. *brucei*, as it uses a highly sophisticated strategy of antigenic variation of a surface Variant Surface Glycoprotein (VSG) coat allowing effective escape from the mammalian immune system [4, 5]. As individual trypanosomes have thousands of different *VSG* genes and pseudogenes, and new chimeric VSG variants are continuously being generated [6], treatment of HAT has relied on drug treatment rather than immunisation.

Only a limited number of drugs are effective against HAT, of which many are toxic, expensive, or difficult to administer in the field [7]. Pentamidine is used against early stage infection of *T*. *brucei gambiense*, with eflornithine (DFMO) or NECT (nifurtimox-eflornithine combination treatment) also effective against later stages of *T*. *brucei gambiense* infection [8, 9]. Suramin has been used since 1922 against early stage *T*. *brucei rhodesiense*, with the highly toxic arsenical drug melarsoprol effective against the later stages of the disease once parasites have penetrated the blood-brain barrier [8]. A constant concern with this limited number of treatment options is human drug toxicity, as well as the development of parasite resistance. Drug resistant *T*. *brucei* strains are easily generated in the laboratory and have been found in the field [10, 11]. While there is a clear need for new treatments for HAT, the decreasing number of HAT cases has reduced the incentive to develop new drugs.

This increases the attractiveness of ‘repurposing’ drugs which have already undergone clinical trials for use against other human diseases [12–14]. The anti-trypanosomal drug eflornithine is a good example of drug “repurposing”. Originally developed as a drug against cancer, it was subsequently repurposed for use against *T*. *brucei gambiense*, as well as female hirsutism [15]. Similarly the drug tamoxifen, which is effective against estrogen receptor-positive breast cancer, also has efficacy against *Leishmania* [16, 17]. In addition to repurposing drugs, target repurposing can also be used to exploit libraries of small molecules developed against human target molecules. For example, an extensive panel of human kinase inhibitors are currently being investigated for their selective potential against essential *T*. *brucei* kinases [18].

Here, we investigate the efficacy of RNA polymerase I (Pol I) transcription inhibitors as treatment against *T*. *brucei*. Pol I transcribes the ribosomal RNA genes (rDNA), which accounts for up to 60% of transcription in proliferating cells [19]. Tumour cells are highly sensitive to disruption of Pol I transcription [20], while normal cells remain largely unaffected [21]. Tumour cell death upon Pol I transcription inhibition is not due to ribosome depletion, but due to cell “checkpoint” activation [20–22], explaining why in particular tumour cells undergoing uncontrolled cell proliferation are exquisitely sensitive to inhibition of Pol I transcription. Chemical inhibitors of Pol I are therefore currently being investigated for treatment against cancer [20, 23, 24].

African trypanosomes are unicellular eukaryotes which proliferate at high rates within the mammalian bloodstream. They have a particularly high dependency on Pol I transcription, as they transcribe the gene encoding their VSG coat from a Pol I transcribed VSG expression site (ES) transcription unit [25, 26]. VSG is highly essential in *T*. *brucei* both *in vitro* and *in vivo*, and perturbation of VSG synthesis *in vivo* results in very rapid trypanosome clearance within hours [27]. We therefore hypothesised that Pol I transcription inhibitors might represent useful drug leads against *T*. *brucei*.

A number of compounds have been shown to selectively impact on Pol I transcription (referred to here as Pol I inhibitors), including quarfloxin, CX-5461 and BMH-21. Quarfloxin has anti-cancer activity, and using a mouse xenograft model system, a large range of cancer cell lines were shown to be sensitive to it *in vivo* [28]. Quarfloxin was subsequently investigated for its therapeutic potential [28–30], and reached Phase II clinical trials before withdrawal due to problems with bioavailability [31]. The Pol I inhibitor CX-5461 was identified in a small molecule screen of compounds inhibiting transcription [32]. Similar to quarfloxin, CX-5461 prevented proliferation of a broad range of cancer cell lines *in vitro* or solid tumours *in vivo* [21, 33, 34]. This lead to its investigation as an anti-cancer therapy, and it is currently in Phase I clinical trials against breast cancer (Clincaltrials.gov NCT02719977) and hematologic cancer (ANZCTR ACTRN12613001061729)[23, 35]. The small molecule BMH-21 was identified in a small molecule screen targeting the p53 tumour suppressor pathway, and is also considered to have possible therapeutic potential against cancer [36, 37].

Here we show selective sensitivity of *T*. *brucei* compared with human breast epithelial or fibroblast cell lines for the Pol I inhibitors quarfloxin, CX-5461 and BMH-21. Trypanosome sensitivity for these drugs is within the nanomolar range, and at concentrations which are therapeutic against cancer. We show that these Pol I inhibitors specifically target Pol I transcription in *T*. *brucei*, as incubation results in very rapid and specific disappearance of Pol I derived RNA precursor transcripts. In addition, incubation with these compounds leads to Pol I subnuclear structures including the VSG expression site body (ESB) and the nucleolus disassembling. These chemical inhibitors demonstrate that the ESB, like the nucleolus, is a Pol I transcription-seeded subnuclear structure. In addition, these data argue that repurposing these potential cancer chemotherapy agents could potentially provide therapeutic potential against African trypanosomes.

## Materials and methods

### Ethics statement

No patient material was used. No animal experiments were performed. All experiments were performed *in vitro* with the established laboratory strain *Trypanosoma brucei* 427.

### Trypanosome strains and culturing

The laboratory bloodstream form strain *Trypanosoma brucei* 427 was cultured *in vitro* according to [38]. The *T*. *brucei* SM or “single marker” cell line [39] or the SM derived S16_221Pur cell line (containing a construct with a puromycin resistance gene inserted behind the active *VSG221* ES promoter) were used for all *in vitro* cytotoxicity and proliferation assays, and were cultured in drug free media for at least 48 hours prior to treatment. The *T*. *brucei* S16221PuroGFP cell line was used for RNA precursor transcript analysis [40, 41]. Selection on puromycin maintained active transcription of the *VSG221* ES in these cell lines. For the immunofluorescence analyses, the *T*. *brucei* TY-YFP-RPA2 cell line was generated through transfection of pEnT5H-Y:NLS:RPA2 (gift of the Gull lab) into *T*. *brucei* SM cells [42]. This resulted in a single RPA2 allele (second largest Pol I subunit) endogenously tagged with Yellow Fluorescence Protein (YFP) at the N-terminus. For analysis of VEX1 foci, the *T*. *brucei* S16_221Pur cell line was transfected with the pNATVEX1^x12myc^ construct (gift of the Horn lab) [43]. This resulted in the addition of a 12x myc C-terminal epitope tag to VEX1.

### Pol I inhibitors and proliferation, wash-out and cytotoxicity assays

RNA polymerase I inhibitors used were CX-5461 (Ross Hannan, ANU, Australia) [32], quarfloxin (CX-3543) (Adooq Bioscience) [28] or BMH-21 (Sigma) [36], with suramin (Sigma) used as a lethality control. Stock solutions of CX-5461 (10mM) were made up in 50 mM NaH_2_PO_4_ (pH 7.0). Quarfloxin (1 mM) and BMH-21 (1 mM) were dissolved in DMSO (dimethyl sulfoxide) (Sigma ≥99.9%). A 10 mM solution of suramin was made in nuclease free water immediately prior to use. All compounds were diluted directly in HMI-9 media immediately before use.

For proliferation assays, *T*. *brucei* SM was treated with inhibitors, and densities were determined using a Neubauer haemocytometer. Growth curves were repeated minimally in triplicate. For wash-out assays, *T*. *brucei* SM was treated with various concentrations of inhibitors. After two hours the cells were washed, resuspended in drug free HMI-9 medium, and cell proliferation was monitored using a haemocytometer. For the Alamar Blue *in vitro* cytotoxicity assay [44], 200 μl samples of *T*. *brucei* SM cells (2 x 10^3^ cells ml^-1^) were plated in 96 well plates, and incubated for 72 hours with two fold serial dilutions of the inhibitors. At 72 hours, 20 μl of Resazurin (0.125 mg ml^-1^) (Sigma) was added, and the parasites were incubated for a further 18 hours. Fluorescence was measured using a Tecan infinite plate reader (excitation at 530 nm, emission at 585 nm). The change in fluorescence (minus the chemical only control) was plotted as a function of the concentration of chemical compound using the sigmoidal dose-response (variable slope) algorithm using GraphPad Prism version 5.

For the mammalian cell toxicity assays, either the spontaneously immortalised Michigan Cancer foundation (MCF10A) human breast epithelial cell line was used [45], or the BJ3 cell line, which is a human foreskin fibroblast cell line immortalized with the h-Tert catalytic subunit of telomerase [46]. Cells were seeded in 96-well plates, and after 48 hours each well was treated in quintuplicate using ten different concentrations of CX-5461, quarfloxin, BMH-21 or suramin. After incubation for 48 hours, the cells were imaged and cell confluence was calculated using an IncuCyte ZOOM (Essen Biosciences). Cell confluence was normalised to a drug vehicle only control which was set to 100%. An Alamar Blue assay was also performed on the MCF10A cell line in the presence of the different Pol I transcription inhibitors. Here the Alamar Blue readout was also normalised to the vehicle only control. Experiments were performed as three biological replicates with the exception of the Incucyte data for MCF10A in the presence of BMH-21 and doxycycline which were obtained in duplicate. The dose response curves and IC50s were calculated using GraphPad Prism.

### Immunofluorescence microscopy

Trypanosomes were fixed in 2% paraformaldehyde, permeabilised with 0.1% NP-40 for 5 minutes at room temperature before incubation for one hour with an antibody against the nucleolar marker L1C6 [47] or anti-myc tag antibody (clone 4A6, EMD Millipore). Slides were subsequently incubated with a goat anti-mouse secondary antibody coupled to Alexa-594 (Molecular Probes), before mounting in Vectashield containing DAPI (Vector Laboratories). Microscopy was performed using a Zeiss Imager.M1 microscope equipped with a Zeiss AxioCam MRm camera and Axio Vision Rel 4.8 software. A Z-stack of images was taken at 200 nm intervals, and the images from the individual channels were processed using Image J software. For quantitation of ESB signal using YFP, VEX1 signal using the anti-myc tag antibody or nucleolar signal using the L1C6 antibody, the exposure time and contrast was uniformly adjusted across all conditions. Quantification of ESB and nucleolar signal was carried out using compressed stacks of all appropriate channels in which the images acquired on the Z-axis were analysed. The ESB and nucleolar status was recorded for a total of approximately 100 cells in G1 (two biological replicates of approximately 50 cells) for each condition.

### Transcript analysis

RNA transcript analysis was performed using quantitative reverse transcription PCR (qRT-PCR). Total RNA was isolated using the RNeasy kit (Qiagen) and DNase treated with TURBO DNA-free kit (Invitrogen). Reverse transcription was performed using 100 ng RNA as a template for cDNA synthesis using random hexamer primers (Promega) and the Omniscript RT kit (Qiagen). qPCR was performed on the 7500 Fast Real-Time PCR system (Life Technologies) using Brilliant II SYBR Green QPCR Low ROX Master Mix (Agilent Technologies). DNase treated RNA without reverse transcriptase was used as a control. The amplification conditions for each primer pair were optimised, and the primer sequences are listed in S1 Table. Transcript levels were normalised against actin mRNA, and plotted as relative change relative to the zero hour time point.

### Statistical analysis

Where indicated, the data was analysed using the Student’s t-test (paired, two-tailed) (GraphPad Prism version 5). Data was considered ‘significant’ where P = 0.01-0.05 (*), ‘very significant’ where P = 0.001-0.01 (**) or ‘extremely significant’ where P = <0.001 (***).

## Results

### Efficacy of RNA polymerase I inhibitors against bloodstream form *Trypanosoma brucei*

**Fig 1:**
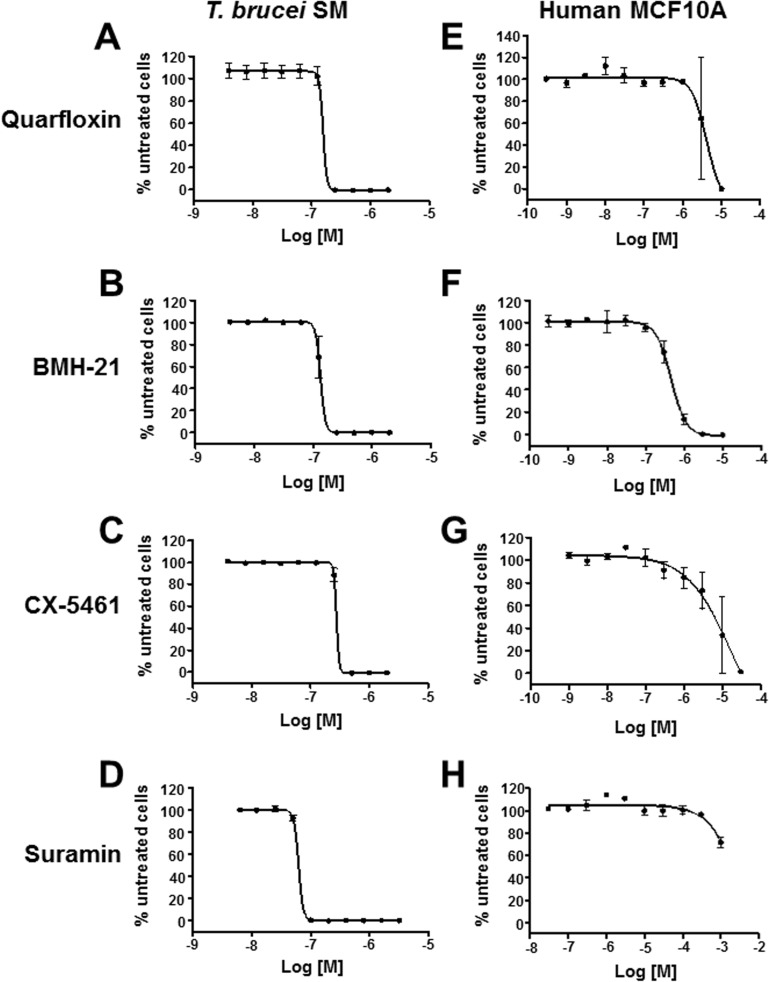
RNA polymerase I (Pol I) transcription inhibitors selectively inhibit proliferation of bloodstream form *Trypanosoma brucei*. An Alamar Blue *in vitro* cytotoxicity assay was used to determine sigmoidal dose response curves of *T*. *brucei* SM (A-D) or the MCF10A human breast epithelial cell line (E-H) incubated with the Pol I inhibitors quarfloxin (A, E), BMH-21 (B, F), CX-5461 (C, G) or the anti-trypanosomal agent suramin (D, H) [44]. The mean percentage of signal relative to the negative control (vehicle without drug) from three biological replicates is plotted with the standard deviation indicated with error bars.

Bloodstream form *T*. *brucei* utilises RNA polymerase I (Pol I) to transcribe rRNA within the nucleolus, as well as the active *VSG* ES in the ESB [48–50]. Blocking VSG synthesis triggers a precytokinesis arrest within one cell division, and very rapid trypanosome clearance *in vivo* [27]. The vital role of this multi-functional RNA polymerase I in African trypanosomes therefore makes it an appealing drug target. In light of this, we tested the RNA Pol I inhibitors quarfloxin (CX-3543), BMH-21 and CX-5461 for their efficacy against bloodstream form *Trypanosoma brucei*. We generated dose response curves using an Alamar Blue *in vitro* cytotoxicity assay, with the anti-trypanosomal agent suramin as a lethality control (Fig 1A–1D) [28, 32, 36, 51]. We found that *T*. *brucei* showed susceptibility to each of these Pol I inhibitors in the nanomolar range. *T*. *brucei* was most sensitive to BMH-21 with an IC50 for proliferation of 134 ± 8 nM (Table 1). Next most effective were quarfloxin with an IC50 of 155 ± 9 nM and CX-5461 with an IC50 of 279 ± 16 nM. As expected, *T*. *brucei* was susceptible to suramin with an IC50 of 63 ± 5 nM, comparable to the value of 53.0 nM found for *T*. *b*. *rhodesiense* using a similar Alamar Blue assay [51].

**Table 1:** Relative toxicity of Pol I inhibitors in *T*. *brucei* compared with human breast epithelial or fibroblast cells expressed as IC50 (μM ± SD)^a^. The selectivity index for *T*. *brucei* is indicated in brackets.

	Alamar blue		Incucyte assay	
Compound	*T*. *brucei*	MCF10A Human breast epithelial cells	BJ3 human fibroblasts	MCF10A Human breast epithelial cells
**Quarfloxin**	0.155 ± 0.009	4.44 ± 3.29	2.72 ± 0.17	6.24 ± 3.87
		(29x)	(18x)	(40x)
**BMH-21**	0.134 ± 0.008	0.46 ± 0.08	1.36 ± 0.22	0.622 ± 0.34
		(3.4x)	(10x)	(4.6x)
**CX-5461**	0.279 ± 0.016	6.89 ± 4.83	9.78 ± 0.79	7.84 ± 9.8
		(25x)	(35x)	(28x)
**Suramin**	0.063 ± 0.005	1636 ± 317	890 ± 127	1260 ± 90
**Doxycycline**		50.7 ± 33.8		

^a^ *T*. *brucei* cell proliferation was determined using a 72 hour Alamar Blue based cytotoxicity assay, with the IC_50_ values presented as the mean ± standard deviation (SD) for three biological replicates of quadruplicate samples after subtraction of fluorescence from the inhibitor only control. Suramin served as a positive control. Proliferation of the MCF10A human breast epithelial cell line was determined using either a 72 hour Alamar Blue or a 48 hour IncuCyte cell proliferation assay. Doxycycline served as a positive control. Growth inhibition of the Tert immortalised BJ3 human fibroblast cell line was determined using an IncuCyte assay. The IC_50_ values are presented as the mean ± SD.

Toxicity of these compounds was also determined in mammalian cells using an Alamar Blue cytotoxicity assay in a spontaneously immortalised breast epithelial cell line (MCF10A) [45] (Fig 1E–1H). The compound doxycycline was used as a positive control for toxicity. These human epithelial cells were the least susceptible to CX-5461 (IC50 of 6.89 ± 4.83 μM), followed by quarfloxin (4.44 ± 3.29 μM) and BMH-21 (460 ± 80 nM). In parallel, proliferation of these human cells in the presence of Pol I inhibitors was monitored by assessing cell confluence using an IncuCyte ZOOM. This automated system allows monitoring of cells in real-time using live cell imaging, and produced comparable results to the Alamar Blue assay (S1 Fig) (Table 1).

In parallel, the BJ3 human foreskin fibroblast cell line that had been immortalised with hTert (the catalytic subunit of telomerase) was also tested for sensitivity to these compounds [46] (S2 Fig)(S3 Fig). The IC50 values corresponding to either growth arrest or cell death shown in S2 Fig were obtained by observing the cell images (S3 Fig) and determining the point where the specific dose curve plateaus at the higher concentrations. If the cells were morphologically sound, then the dose curve represented growth arrest of the cells. If the cells were dead, then the dose curve represented cell death. The IC50 values for cell death were used to calculate the selectivity index scores. This was done as the mammalian cells used in the dose response assays were intended to approximate normal quiescent human cells, hence the IC50 values for growth arrest are not relevant.

Similar to the breast cancer cells, these human fibroblasts were the least susceptible to CX-5461 (IC50 of 9.78 ± 0.79 μM), followed by quarfloxin (2.72 ± 0.17 μM) and BMH-21 (1.36 ± 0.22 μM) (Table 1). If one therefore compares the relative selectivity of these compounds for *T*. *brucei*, CX-5461 is the most effective compound, with *T*. *brucei* 25-35 fold more sensitive than human cells. *T*. *brucei* is 18-40 fold more sensitive to quarfloxin and 3-10 fold more sensitive to BMH-21. As expected, the trypanocide suramin showed minimal toxicity to mammalian cells (IC50 of 1636 ± 317 μM).

**Fig 2:**
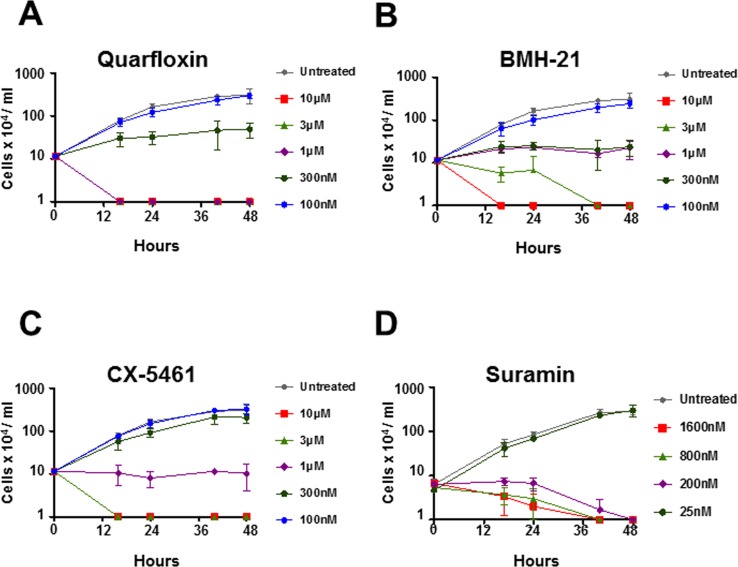
Pol I transcription inhibitors kill bloodstream form *Trypanosoma brucei* in a time and dose dependent manner. Cell proliferation assays were performed in which *T*. *brucei* SM cells were exposed to a range of concentrations of Pol I inhibitors including quarfloxin (A), BMH-21 (B) or CX-5461 (C) using the anti-trypanosomal agent suramin (D) as a control. The graph shows the cell density of treated parasites in comparison with the untreated *T*. *brucei* SM line at 0, 16, 24, 40 and 48 hours. The mean of four biological replicate experiments for the Pol I inhibitors, or three replicates for the suramin control are shown with standard deviation indicated with error bars.

We next investigated the effect of these Pol I inhibitors on *T*. *brucei* proliferative growth over 48 hours (Fig 2). The drug concentrations of quarfloxin, BMH-21 or CX-5461 that resulted in suppression of proliferative growth in *T*. *brucei* were 300 nM, 300 nM and 1 μM respectively (Fig 2A–2C), with suramin serving as a positive control (Fig 2D). The impact of these Pol I inhibitors on *T*. *brucei* cell growth was rapid, and evident within two cell divisions.

**Fig 3:**
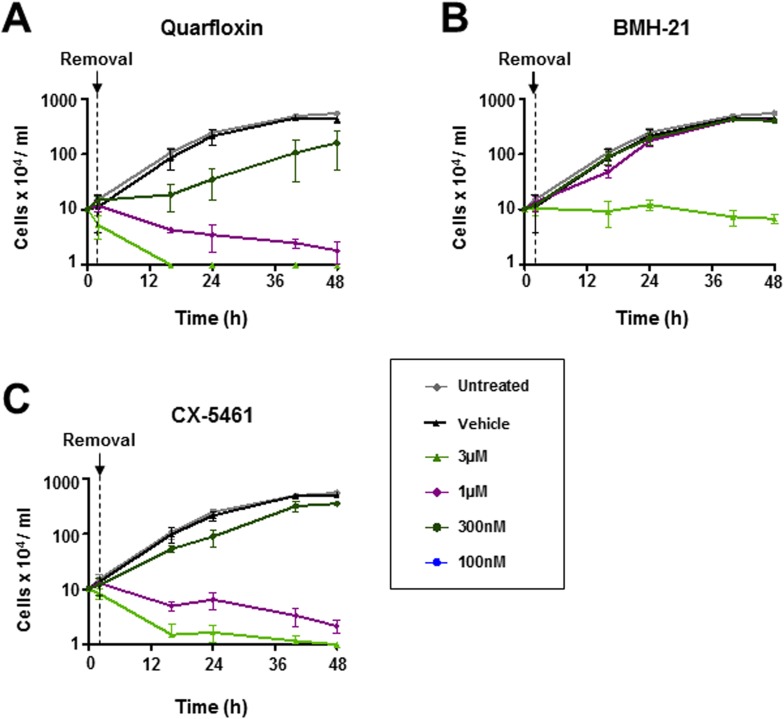
Irreversible inhibition of *T*. *brucei* growth after incubation with quarfloxin or CX-5461 as determined using wash-out assays. *T*. *brucei* SM cells were treated with various concentrations of quarfloxin (A), BMH-21 (B) or CX-5461 (C) for two hours, washed, diluted into fresh media and cell growth was monitored. Untreated cells, and parasites incubated with the appropriate drug vehicle (DMSO for quarfloxin and BMH-21) or NaH_2_PO_4_ (for CX-5461) were used as controls. The mean of three biological replicates is plotted with the standard deviation indicated with error bars.

Under therapeutic conditions effective drug concentrations in the blood typically fluctuate through time. We therefore tested the reversibility of these Pol I transcription inhibitors on the inhibition of *T*. *brucei* proliferation using wash-out assays. *T*. *brucei* was incubated with various concentrations of these inhibitors for two hours. Cells were subsequently washed and resuspended in drug-free medium, before cell proliferation was monitored (Fig 3). Incubation of *T*. *brucei* with quarfloxin and CX-5461 for two hours still resulted in significantly reduced proliferation at similar concentrations of drug (1 μM) even after the wash-out was performed. This indicates that quarfloxin and CX-5461 were working irreversibly. In contrast, wash-out of the BMH-21 inhibitor at all except for the highest concentration (3 μM) resulted in restored *T*. *brucei* growth. This indicates reversibility in how this compound is inhibiting trypanosome growth, which could potentially limit its use as a therapeutic agent.

### Treatment with Pol I inhibitors leads to selective reduction in Pol I transcription

**Fig 4:**
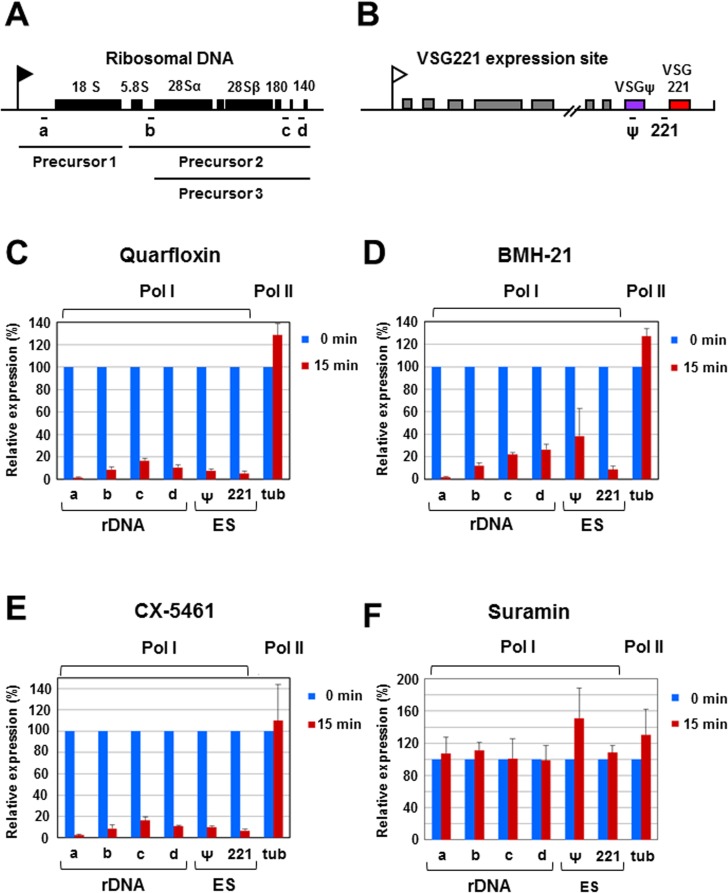
Rapid and specific inhibition of Pol I transcription by Pol I inhibitors in bloodstream form *T*. *brucei*. (A) Schematic of the ten kilobase pair *T*. *brucei* ribosomal DNA (rDNA) transcription unit with the rDNA promoter indicated with a black flag, and the rRNA genes with black boxes. Different rRNA precursor transcripts are shown below according to [52], with the qPCR primers used indicated with letters. The first rRNA cleavage produces rRNA Precursor 1 (3.4 kb) and Precursor 2 (5.6 kb) transcripts. Subsequently, Precursor 2 is cleaved to generate Precursor 3 (5.0 kb) [52]. (B) Schematic of the sixty kilobase pair *VSG221* expression site with the promoter indicated with a white flag. Various expression site associated genes are indicated with grey boxes, and the telomeric *VSG* pseudogene (ψ) and the *VSG221* gene indicated with coloured boxes with relevant primers indicated below. These transcription units are not drawn according to scale. (C) Rapid and specific inhibition of Pol I transcription in the presence of quarfloxin. The *T*. *brucei* S16221PuroGFP cell line was incubated with 1 μM quarfloxin for 15 minutes. RNA precursor transcripts analysed were either from the Pol I transcribed rDNA (primer pairs a-d) or the active *VSG221* ES. ES transcripts analysed corresponded to a *VSG* pseudogene (ψ) or a *VSG221* precursor transcript (221). In comparison, levels of precursor transcript from the Pol II transcribed alpha-beta tubulin locus (tub) remained unaffected. RNA was normalised against actin mRNA, levels of which remained unchanged by the different Pol I inhibitors. Results shown are the mean of three biological replicates with standard deviation indicated with error bars. (D) As in (C), only cells were incubated in 1 μM BMH-21. (E) As in (C), only cells were incubated in 1 μM CX-5461. (F) As in (C), only cells were incubated with 800 nM suramin.

As these Pol I inhibitors dramatically affected *T*. *brucei* proliferation, we next investigated if Pol I transcription of the rDNA or VSG ES was selectively inhibited (Fig 4A and 4B). The rDNA in *T*. *brucei* is transcribed as an approximately ten kilobase transcription unit, with the pre-rRNA precursor transcripts subsequently undergoing rapid processing reactions (Fig 4A) [52]. After incubation of *T*. *brucei* with the Pol I inhibitors, we determined the abundance of these unstable rRNA precursors using qRT-PCR as an indirect method of rRNA synthesis rates as performed previously [53]. Incubation with 1 μM quarfloxin, resulted in a dramatic reduction in pre-rRNA precursors within 15 minutes (Fig 4C), and pre-rRNA Precursor 1 transcript levels were reduced by 98.5 ± 0.66% (primer a), and rRNA Precursor 2 decreased by 91.3 ± 2.6% (primer b). Comparable degrees of repression of rDNA transcription were seen using primer pairs c and d, which detect both rRNA Precursors 2 and 3, where levels were also drastically reduced within fifteen minutes by 83.4 ± 2.1% or 89.5 ± 2.7% respectively.

The VSG221 ES is a highly active Pol I transcription unit in the bloodstream form of *T*. *brucei*. We investigated the repression of transcripts derived from regions of the ES which could be expected to be unstable, as they are encoded by intergenic regions or pseudogenes. We monitored the presence of a *VSG221* precursor transcript using a primer pair 520 bp upstream of the *VSG221* gene (Fig 4B). This *VSG221* precursor transcript was reduced by 94.5 ± 1.9% after 15 minutes incubation with quarfloxin (Fig 4C). A single copy *VSG* pseudogene (ψ) is located approximately 4.7 kb upstream of the telomeric *VSG221* in the approximately 60 kilobase *VSG221* expression site [49]. Even when the *VSG221* expression site is transcriptionally active, only very low levels of this *VSG* pseudogene transcript are present, presumably as a consequence of rapid degradation by nonsense mediated decay [54]. After incubation of *T*. *brucei* with quarfloxin for 15 minutes, levels of *VSG* pseudogene transcript were reduced by 92.3 ± 1.6%.

The rapid disappearance of these RNA precursors indicates that both the Pol I derived pre-rRNA as well as the Pol I derived *VSG* expression site encoded RNA precursor transcripts for *VSG221* and the *VSG* pseudogene have very short half-lives of approximately three or four minutes. Precursor transcripts from the tubulin gene cluster are also highly unstable, and have been estimated to have a half-life of about one minute [55, 56]. However, treatment with quarfloxin for fifteen minutes did not result in a reduction in the levels of Pol II derived tubulin precursor transcript (129 ± 10.3% normal). Actin mRNA has been estimated to have a half-life of about 30 minutes in bloodstream form *T*. *brucei* [57]. We did not find that actin mRNA levels were affected by any of the inhibitors used in this study, and therefore the qPCR results were normalised using this transcript. These experiments therefore provide evidence for the selective inhibition of Pol I transcription in *T*. *brucei* with quarfloxin, which was highly significant (*** P = <0.001) in all cases.

Similar striking repression of Pol I transcription was observed after incubation of *T*. *brucei* with 1 μM BMH-21 for fifteen minutes (*** P = <0.001) (Fig 4D). In addition, incubation with 1 μM CX-5461 inhibitor for 15 minutes produced similar results to the other two inhibitors with specific knockdown of Pol I transcripts (*** P = <0.001 in all cases) (Fig 4E). To confirm that the selective reduction in Pol I derived precursor transcripts was not simply a consequence of cell lethality following treatment with the Pol I inhibitors, cells were treated for 15 minutes with 800 nM suramin (Fig 4F). Despite eventually causing cell death, this suramin treatment did not lead to reduced levels of any of these transcripts. In summary, these data show that incubation of *T*. *brucei* with all three Pol I inhibitors results in robust and rapid inhibition of Pol I transcription.

### Incubation with Pol I inhibitors leads to loss of the ESB and disassembly of the nucleolus

One of the hallmarks of blocking Pol I transcription is disassembly of the nucleolus as its integrity is dependent on rRNA synthesis [58]. We therefore monitored the disappearance of intact nucleoli as well as the ESB using immunofluorescence microscopy after incubation of *T*. *brucei* with the different Pol I inhibitors. The *T*. *brucei* RNA polymerase I complex contains at least twelve subunits, of which RPA2 is the second largest subunit [59, 60]. RPA2 has previously been endogenously tagged at the N-terminus with Yellow Fluorescence Protein (YFP) in bloodstream form *T*. *brucei*, allowing visualisation of the Pol I complex in both the nucleolus and the ESB [42]. In these experiments the ESB was visible in 67% ± 5% of nonmitotic cells [42]. We generated *T*. *brucei* expressing YFP tagged RPA2 from the endogenous RPA2 locus in the bloodstream form of the parasite, as described previously by Daniels *et al* [42]. As expected, there was no growth reduction in these cell lines, and the YFP-RPA2 subunit showed the expected subnuclear location for a Pol I subunit.

**Fig 5:**
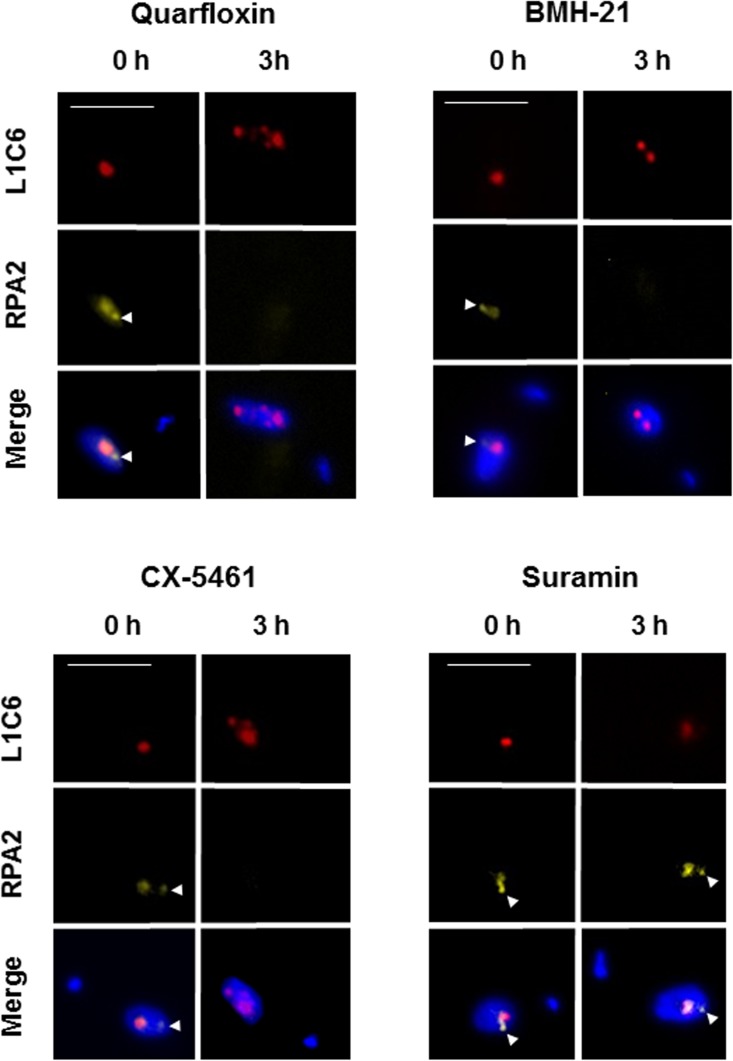
Incubation of *T*. *brucei* with Pol I transcription inhibitors leads to rapid disappearance of the Pol I Expression Site Body (ESB) and fragmentation of the nucleolus. Immunofluorescence analysis of *T*. *brucei* TY-YFP-RPA2 incubated with 3 μM quarfloxin, BMH-21, CX-5461 or 800nM suramin for three hours (h). The panels show representative *T*. *brucei* cells in the G1 cell cycle stage with DNA visualised with the DNA stain DAPI, and the nucleolus with the L1C6 nucleolar marker. The Pol I complex can be visualised using Yellow Fluorescence Protein (YFP), as one allele of the RPA2 gene (second largest Pol I subunit) is epitope tagged. Signal corresponding to the extra-nucleolar ESB is indicated with a white arrow head. Scale bar indicates 5 μm.

**Fig 6:**
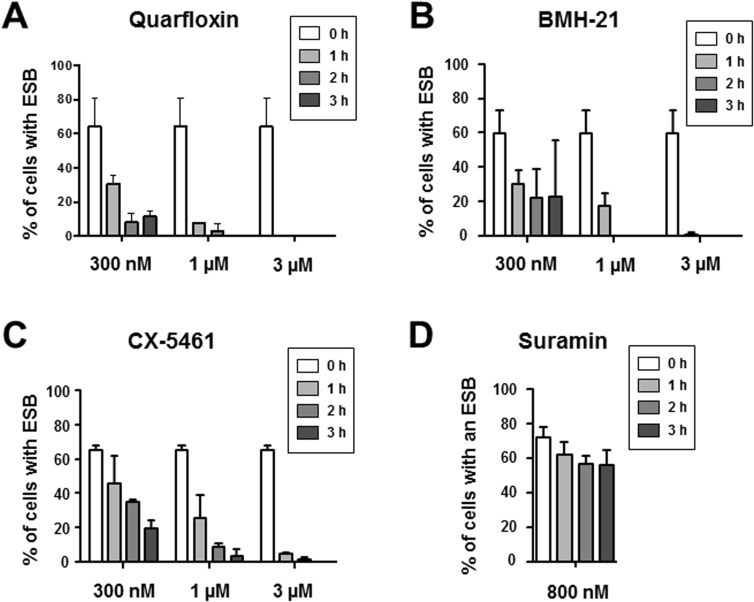
Time dependent disappearance of the Pol I Expression Site Body (ESB) in *T*. *brucei* incubated with Pol I transcription inhibitors. Quantitation of ESB presence in *T*. *brucei* cells in the G1 cell cycle stage incubated with various concentrations of quarfloxin (A), BMH-21 (B), CX-5461 (C) or 800 nM suramin (D). The percentage (%) of cells with an ESB after incubation for the time indicated in hours (h) is shown. A total of 100 cells (two biological replicates of ~50 cells) was analysed with the standard deviation indicated with error bars.

Using the DNA stain DAPI, the *T*. *brucei* nucleus can be visualised as a large stained focus, with a smaller focus indicating the kinetoplast (mitochondrial) DNA. The Pol I subunit RPA2 was visualised using YFP fluorescence, and the location of the nucleolus was confirmed using the L1C6 nucleolar marker [47]. As expected, YFP-RPA2 was enriched in the nucleolus in all of the cells, and in a small extra-nucleolar ESB structure in 64 ± 17% of the cells (Fig 5, Fig 6) as shown earlier [42]. We next investigated the effect of the Pol I inhibitors on the presence of the Pol I complex. As one of the hallmarks of blocking rRNA transcription is disintegration of the nucleolus [58], an antibody against the nucleolar marker protein L1C6 was used to visualise this. *T*. *brucei* cells in the G1 stage of the cell cycle have one nucleolus, and incubation of *T*. *brucei* with quarfloxin (3 μM) resulted in disintegration of this nucleolus into multiple L1C6 containing spots. In addition, there was loss of Pol I signal from the ESB, as well as from the nucleolar remnants (Fig 5). Incubation with 3 μM BMH-21 and CX-5461 also resulted in similar ESB loss and nucleolar disintegration (although not complete loss) in *T*. *brucei* (Fig 5). In contrast, incubation with lethal concentrations of the trypanocide suramin (800 nM) did not impact on distribution of Pol I or result in changes in ESB or nucleolar morphology.

We next quantitated the effect of different concentrations of quarfloxin on the presence of the ESB. Incubation with 300 nM quarfloxin for one hour resulted in a reduction in ESB positive cells from 64 ± 17% to 30.5 ± 5%, while in 1 μM quarfloxin only 7.5 ± 0.7% of the cells were ESB positive after one hour (Fig 6A). No cells contained an ESB after one hour of incubation with 3 μM quarfloxin. A similar analysis was performed with BMH-21, where very similar results were obtained (Fig 6B). Incubation with a range of concentrations of CX-5461 also resulted in a time and dose dependent decrease in presence of the ESB (Fig 6C). We used suramin in order to establish that the observed loss of the ESB in these experiments was not simply a consequence of subnuclear architecture falling apart in dying cells. Suramin rapidly kills *T*. *brucei* at a concentration of 800 nM (Fig 2). After one to three hours incubation with 800 nM suramin, there was only a marginal reduction in ESB positive cells (Fig 6D).

**Fig 7:**
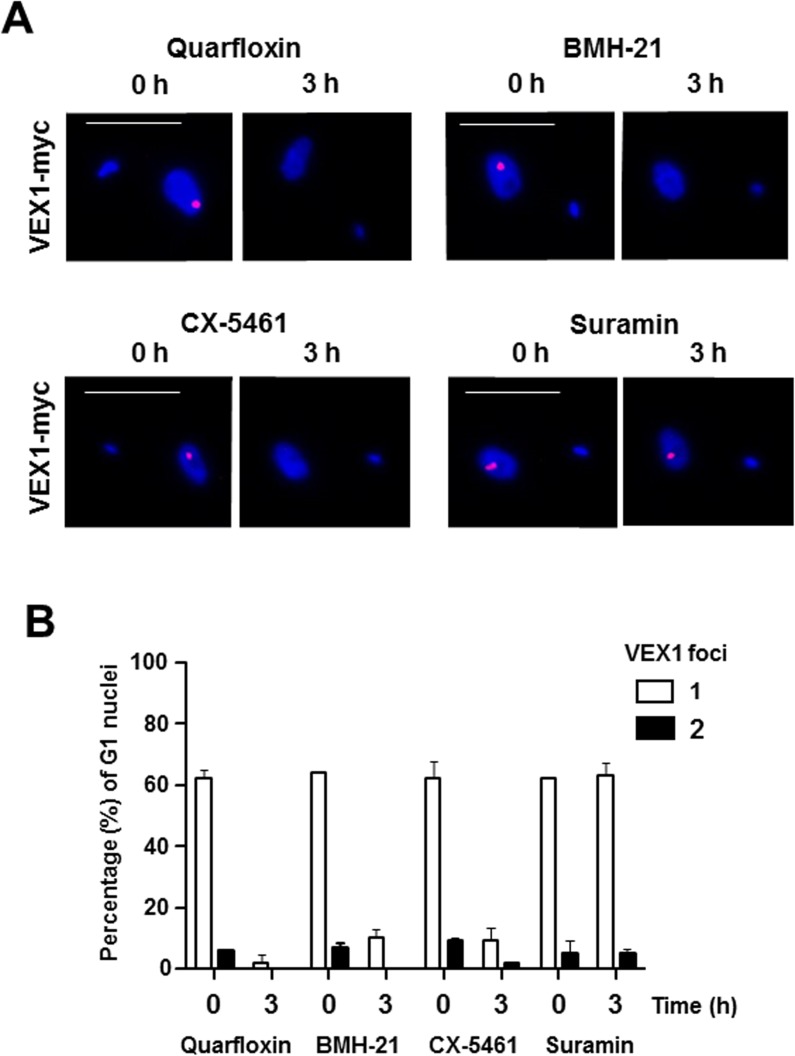
Incubation of *T*. *brucei* with Pol I transcription inhibitors results in a dramatic decrease in VEX1 foci. (A) Immunofluorescence analysis of *T*. *brucei* S16_221PurVEX1x12myc incubated with 3 μM quarfloxin, BMH-21, CX-5461 or 800 nM suramin for three hours (h). The panels show representative cells in the G1 cell cycle stage. DNA is stained with DAPI and the myc-epitope tagged VEX1 protein visualised with an anti-myc antibody and Alexa954 secondary. Scale bar indicates 5 μM. (B) Quantification of VEX1 foci present in nuclei of *T*. *brucei* in the G1 cell cycle stage (1K1N) at 0 hours or after a three hour incubation with 3 μM quarfloxin, BMH-21, CX-5461 or 800 nM suramin. The mean percentage (%) of G1 cells with either one or two VEX1 foci per nucleus (two biological replicates of 50 cells) is plotted with the standard deviation indicated with error bars.

Recently, the VEX1 protein has been discovered to play a role in ES regulation in bloodstream form *T*. *brucei*, although how this operates mechanistically remains unclear [43]. In bloodstream form *T*. *brucei* VEX1 associates with the ESB, serving as a marker for this subnuclear structure. We investigated the effect of incubation with Pol I transcription inhibitors on VEX1 localisation. We incubated bloodstream form *T*. *brucei*, which had an endogenous copy of VEX1 epitope tagged with myc, with 3 μM quarfloxin, BMH-21 or CX-5461 (Fig 7). Similar to as observed with YFP tagged RPA2, VEX1 signal was drastically reduced after incubation of the cells with all three Pol I transcription inhibitors. In contrast, although incubation with the trypanocide suramin resulted in severe reduction in trypanosome growth no specific disintegration of the ESB was observed as visualised using epitope tagged VEX1 (Fig 7). These results support the observation that the ESB (as visualised using VEX1) is a transcription nucleated structure.

**Fig 8:**
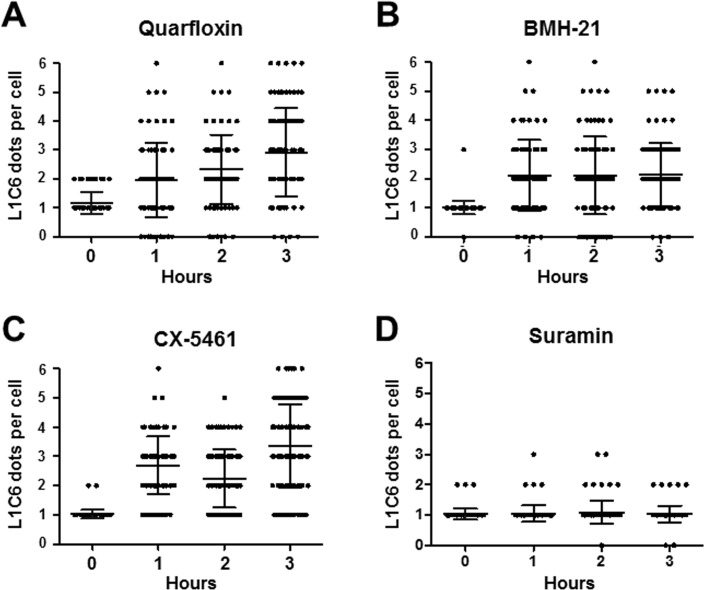
Incubation of *T*. *brucei* with Pol I transcription inhibitors leads to rapid disintegration of nucleoli. The nucleolus can be visualised with the L1C6 nucleolar marker as one spot in *T*. *brucei* in the G1 cell cycle stage. Nucleoli rapidly disintegrate into multiple L1C6 positive spots after incubation of cells with 3 μM quarfloxin (A), BMH-21 (B), CX-5461 (C) or 800 nM suramin (D). Each dot indicates a cell, where the number of L1C6 positive spots is indicated. The mean indicated with a line, and standard deviation with error bars. A total of 100 cells was analysed (two biological replicates of ~50 cells).

Similar to the Pol I ESB body, the structure of the Pol I nucleolar structures rapidly disintegrated (but did not disappear entirely) within one hour in cells incubated with these Pol I inhibitors. Incubation with 3 μM quarfloxin, BMH-21 and CX-5461 universally resulted in nucleoli fragmenting into multiple foci as monitored using the nucleolar marker L1C6 (Fig 8). Again, incubation with suramin did not lead to significant changes in structure of the nucleoli.

We do not know if these Pol I transcription inhibitors operate mechanistically in a similar fashion in *T*. *brucei* compared with mammalian cells. In the case of BMH-21, in human cells BMH-21 blocks Pol I transcription, resulting in degradation of the Pol I large subunit [37]. We investigated if incubation of *T*. *brucei* with increasing concentrations of BMH-21 resulted in similar degradation of the *T*. *brucei* RPA2 Pol I subunit. However even after incubation of *T*. *brucei* TY-YFP-RPA2 with 3 μM BMH-21 for one hour, we did not see evidence for degradation of the RPA2 Pol I subunit (S4 Fig). As BMH-21 intercalates with DNA, it is possible that this DNA binding activity disrupts Pol I transcription in *T*. *brucei* in a different fashion to mammals.

Collectively, these data show that bloodstream form *T*. *brucei* is highly sensitive to Pol I inhibitors compared with the human host. Moreover, Pol I inhibitors rapidly and selectively inhibit Pol I transcription in *T*. *brucei*, leading to loss of the ESB and fragmentation of the nucleolus. Together, these experiments provide evidence that the ESB within *T*. *brucei* is fundamentally a Pol I transcription nucleated structure.

## Discussion

Rapidly proliferating cells require high levels of transcription of the Pol I transcribed rDNA, and chemical inhibitors of this Pol I transcription are effective against cancer [20, 21, 28, 33, 37]. Pol I not only transcribes the rDNA in bloodstream form *T*. *brucei*, but also the highly essential *VSG* gene. *T*. *brucei* is therefore particularly reliant on high levels of Pol I transcription, as even minor perturbations of VSG synthesis would be disastrous for parasite survival *in vivo* [27].

Here, we show that three established mammalian Pol I transcription inhibitors including CX-5461, quarfloxin and BMH-1 are selectively toxic for *T*. *brucei*. The most selective Pol I inhibitors against *T*. *brucei* are CX-5461 with an IC50 of 279 nM and quarfloxin with an IC50 of 155 nM. *T*. *brucei* is therefore 25-35 fold more susceptible to CX-5461 or 18-40 fold more susceptible to quarfloxin compared with the spontaneously immortalised MCF10A human breast epithelial cell line or the immortalised BJ3 human fibroblast cell line. This differential sensitivity could potentially provide a “therapeutic window” to treat a human trypanosome infection using these compounds. Although in *T*. *brucei* BMH-21 has a lower IC50 concentration (134 nM) compared with CX-5461 or quarfloxin, BMH-21 is also more toxic to human cells and there is only 3-10 fold selectivity for *T*. *brucei*. The selective toxicity of 25-35 fold for CX-5461 and 18-40 fold for quarfloxin in *T*. *brucei* compared with human cells meets DNDI criteria for drug screening for Kinetoplastid diseases where the minimum selectivity index is ≥ 10 (Ioset *et al* at: www.dndi.org/2009/media-centre/scientific articles). This makes Pol I inhibitors intriguing drug leads. However, further studies including structure-activity relationship (SAR) studies would clearly be required to optimise both their potency and their selectivity against *T*. *brucei*.

We show that all three Pol I inhibitors selectively and rapidly block Pol I transcription, with rRNA precursor transcripts in some cases reduced by 98%, and VSG expression site derived precursor transcripts reduced by 94%. In contrast, we saw no reduction in levels of precursor transcript from the Pol II transcribed tubulin transcription unit. Incubation of *T*. *brucei* with these three Pol I inhibitors lead to disappearance of Pol I signal within the nuclei within one hour. In addition, within one hour there was disappearance of the ESB (as visualised using RPA2 or VEX1) and rapid disintegration of the nucleolus, which shattered into small dots as visualised using an antibody for the nucleolar protein L1C6.

It has been demonstrated that nucleoli are ‘Pol I transcription-seeded’ structures which require active transcription for maintenance of their structure [58, 61]. This transcription needs to be specifically mediated by Pol I, as nucleoli do not form if rDNA is transcribed by Pol II [62]. The fact that the nucleoli in *T*. *brucei* rapidly disassemble after incubation of the cells with specific Pol I inhibitors, argues that nucleoli are also Pol I transcription-nucleated structures in African trypanosomes. As well as disintegration of the nucleoli, incubation with all three Pol I inhibitors also leads to a very striking and rapid disappearance of the ESB. After one hour incubation with 3 μM BMH-21 or CX-5461, only 1 ± 1.4% or 4.5 ± 0.7% of the cells respectively still had an ESB. After a one hour incubation with 3 μM quarfloxin, none of the cells had an ESB. Similar rapid disappearance of the ESB in the presence of Pol I inhibitors was observed if the ESB was visualised using VEX1. These results would therefore suggest that the *T*. *brucei* ESB similar to the nucleolus, requires active Pol I transcription for its maintenance.

The active *VSG* gene is transcribed at a high rate from a single active telomeric *VSG* expression site locus, allowing the cell to produce vast amounts of *VSG* transcript (approximately 10% total mRNA) from a single copy *VSG* gene. This highly efficient *VSG* transcription, as well as rapid processing of the abundant *VSG* mRNA is presumably facilitated by the presence of the *VSG* expression site within the ESB subnuclear structure. The ESB has been proposed to function as a specialised factory assembled on the active *VSG* expression site, containing high concentrations of Pol I transcription factors as well as the RNA processing machinery necessary for efficient expression of *VSG* [50].

It has been postulated that the ESB is a coherent architectural structure, rather than simply a consequence of resident Pol I on the active ES DNA. This is based on the observation that removal of the DNA in DNase I treated methanol fixed cells, still resulted in concentrated localisation of Pol I in an ESB [50]. However, a potential complication in interpreting these experiments could be a possible reduction in motility in Pol I and associated transcription complexes after the methanol precipitation needed for fixation, even in the absence of DNA. Is the ESB a ‘Pol I transcription-seeded’ structure as has been proposed for the nucleolus? Our results would agree with this, and suggest that the ESB is a subnuclear structure which is nucleated around sites of active Pol I transcription, as the ESB rapidly disappears after Pol I transcription is blocked.

The ESB shares some, but not all components with nucleoli. Key Pol I transcription factors including CITFA-7 and the architectural chromatin protein TDP are located in both Pol I subnuclear structures [53, 63]. However, other nucleolar components including fibrillarin as well as the L1C6 nucleolar protein are found in the nucleolus but not the ESB [47, 50]. The only factor that has yet been proposed to be ESB specific is VEX1, although its mode of action in *VSG* control is yet to be determined [43]. Our experiments using Pol I inhibitors show that these two types of Pol I transcription complexes at either the rDNA or the active ES are inhibited in a similar fashion, although there are clearly significant differences in their control.

It is striking that all three of these mammalian Pol I inhibitors appear to specifically inhibit Pol I transcription in *T*. *brucei* despite having differences in their modes of action in mammalian cells. BMH-21 is a DNA intercalator, which binds GC-rich sequences present at high frequency in ribosomal DNA genes [37, 64]. In human cells BMH-21 blocks Pol I transcription elongation, leading to proteasome dependent destruction of RPA194 (largest Pol I subunit), which is correlated with cancer cell killing [37]. In *T*. *brucei*, although there is also rapid disappearance of Pol I subnuclear structures like the nucleoli and the ESB after one hour incubation with 1 μM BMH-21, we did not see evidence for significant reduction in levels of YFP2-RPA2 as monitored by Western blot analysis (S4 Fig). However, despite the presence of unreduced amounts of YFP-RPA2 protein, intercalation of BMH-21 with DNA could still potentially result in disassociation of Pol I complex from the DNA.

In mammalian cells, CX-5461 inhibits Pol I mediated initiation of transcription at the rDNA through interference of SL1 transcription factor binding to the rDNA promoter [33]. *T*. *brucei* does not have an obvious SL1 homologue, and it is still unclear which protein fulfils its function in trypanosomes. It is therefore, not straightforward to test if the mode of action of CX-5461 in *T*. *brucei* is indeed similar to that in mammalian cells. Quarfloxin is a fluoroquinolone which intercalates with DNA, and is thought to preferentially bind G-rich stretches forming G-quadruplexes within the rDNA and at telomeres [29, 30, 65]. Quarfloxin accumulates in the nucleoli, where it selectively interferes with rRNA synthesis at the level of Pol I elongation, leading to reduced levels of rRNA precursor transcript [28]. Although *T*. *brucei* rDNA has G-rich regions, it is unclear if these indeed form G-quadruplexes. *T*. *brucei* telomere repeats have the same sequence (GGGTTA) as those in mammalian cells, and could therefore also be expected to form G-quadruplex structures [65]. As well as interfering with Pol I transcription of the rDNA in *T*. *brucei*, it is possible that quarfloxin also interferes with telomere function as well. A similarity between BMH-21 and quarfloxin is that they intercalate with DNA, particularly in G-rich regions, and it is presumably this feature that is behind their efficacy in *T*. *brucei*. These Pol I inhibitors could therefore provide a new tool for specifically blocking Pol I transcription in African trypanosomes, which should help us dissect how the regulated Pol I mediated transcription of the VSG variant antigen genes is controlled.

Pol I inhibitors are currently being investigated for their suitability for treating cancer. The inhibitor quarfloxin progressed to Phase II clinical trials, but was withdrawn due to problems regarding bioavailability [31]. However, CX-5461 is still under investigation for cancer treatment, and is currently in Phase I clinical trials against breast cancer (Clincaltrials.gov NCT02719977) and haematologic cancer (ACTRN12613001061729). Could these Pol I inhibitors indeed be used to treat human African trypanosomiasis? Pol I is of particular importance for bloodstream form *T*. *brucei* as very high levels of transcription of the single active *VSG* gene are essential to provide the huge amount of VSG protein required to coat this extracellular parasite. As blocking VSG synthesis results in very rapid parasite clearance in infected mice [27], we would predict that targeting *VSG* transcription should allow us to target this particularly vulnerable feature of the parasite. It is likely that even minor perturbation of *VSG* synthesis would result in rapid trypanosome clearance *in vivo*. This could potentially allow the repurposing of a potential anti-cancer agent for treatment of a tropical parasite.

As clinical trials are the main cost in developing a pharmaceutical treatment, one could envisage that repurposing an existing drug could provide an economical means to increase the number of substances that can be used to treat trypanosomiasis [13, 14]. This is particularly an issue as the current relatively small number of annual human African trypanosomiasis cases make the cost of developing new drugs problematic. However, despite some of the complications that would need to be considered regarding potential toxicity of these compounds, our data indicate that these Pol I inhibitors could potentially function as new chemical weapons against human African trypanosomiasis.

## Supporting information

S1 Fig Dose response curves of MCH10A human breast epithelial cells incubated with Pol I transcription inhibitors. (A-D) An Incucyte assay was used to determine dose response curves of MCF10A human breast epithelial cells incubated for 48 hours with a range of concentrations of Pol I transcription inhibitors or the trypanocidal agent Suramin. Dose response curves for quarfloxin (A), BMH-21 (B), CX-5461 (C) or suramin (D) are shown. The mean percentage (%) of cell confluence from three biological replicates is plotted, except in the case of BMH-21, where the results of two biological replicates are shown. The standard deviation is indicated with error bars. (E) Comparison of the IC50 values for Pol I transcription inhibitors determined using either an Incucyte cell confluence proliferation assay or a Resazurin based assay (Alamar Blue).
(TIF)

S2 Fig Dose response curves of immortalised BJ3 human foreskin fibroblasts incubated with Pol I transcription inhibitors. (A-D) Dose response curves of human foreskin fibroblasts immortalised by the addition of h-Tert and incubated for 48 hours with various doses of (A) quarfloxin, (B) BMH-21, (C) CX-5461 or (D) suramin. The mean percentage (%) of cell confluence is plotted from five biological replicates with the standard deviation indicated with error bars. (E) Summary of IC50 values for growth inhibition (where the higher doses prevent cell growth) and the cell death (where the higher doses cause cell death) (n = 5, error bars represent standard deviation).
(TIF)

S3 Fig Representative images of Incucyte assay of immortalised BJ3 human fibroblasts treated with various Pol I inhibitors. Cells were treated with various doses of quarfloxin, BMH-21, CX-5461 or suramin for 48 hours. Cells before treatment are shown (T = 0). Cells were imaged and cell confluence established using an IncuCyte ZOOM. Scale bars correspond to 100 μm.
(TIF)

S4 Fig Incubation of *T*. *brucei* with the Pol I transcription inhibitor BMH-21 does not lead to significant degradation of the RPA2 Pol I subunit. (A) The *T*. *brucei* TY-YFP-RPA2 cell line was incubated with various concentrations of BMH-21 for the time indicated in hours. As a control, *T*. *brucei* was also incubated with 800 nM of suramin. Lysate from 1 x 10^7^ cell equivalents was blotted and probed with anti-TY (BB2) antibody. A band corresponding to *T*. *brucei* RPA2 is shown, as well as a Ponceau S stain of the blot to serve as a loading control. Size markers in kiloDaltons (kDa) are indicated.
(TIF)

S1 Table List of the primers used in the qPCR experiments. The gene location of the primers is shown, as well as the primer names and their DNA sequence (5’-3’).
(PDF)
