# Supplementary figures and images for: Selective inhibition of RNA polymerase I transcription as a potential approach to treat African trypanosomiasis
Source: PLoS Negl Trop Dis. 2017 Mar 6;11(3):e0005432. doi: 10.1371/journal.pntd.0005432 (PMC5354456; doi:10.1371/journal.pntd.0005432)

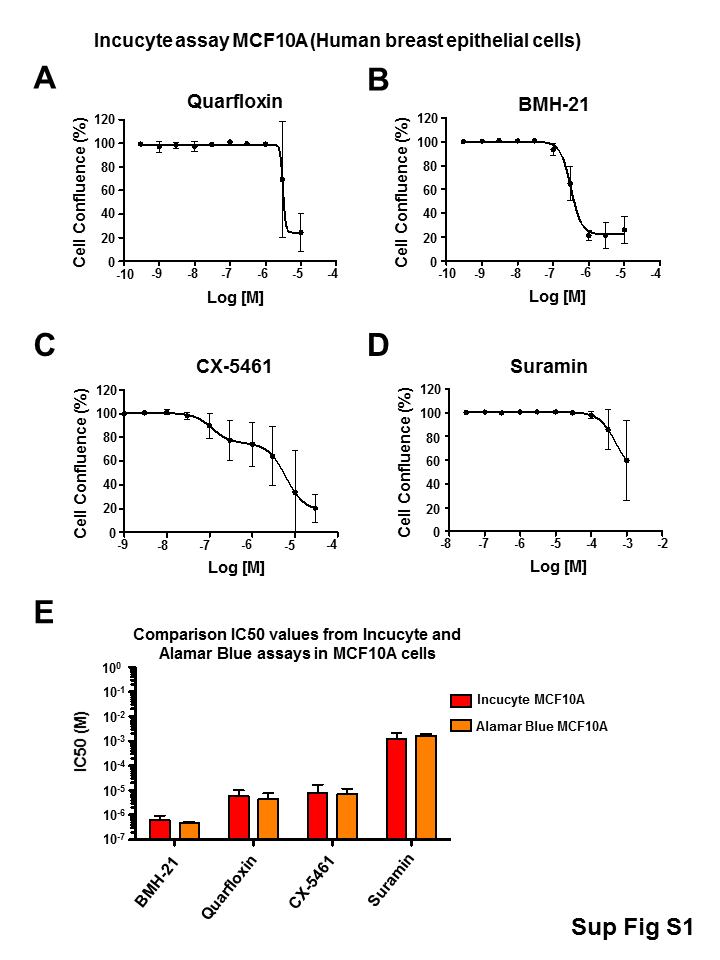

Supplement: S1 Fig — (A-D) An Incucyte assay was used to determine dose response curves of MCF10A human breast epithelial cells incubated for 48 hours with a range of concentrations of Pol I transcription inhibitors or the trypanocidal agent Suramin. Dose response curves for quarfloxin (A), BMH-21 (B), CX-5461 (C) or suramin (D) are shown. The mean percentage (%) of cell confluence from three biological replicates is plotted, except in the case of BMH-21, where the results of two biological replicates are shown. The standard deviation is indicated with error bars. (E) Comparison of the IC50 values for Pol I transcription inhibitors determined using either an Incucyte cell confluence proliferation assay or a Resazurin based assay (Alamar Blue). (TIF) [file pntd.0005432.s001.tif]

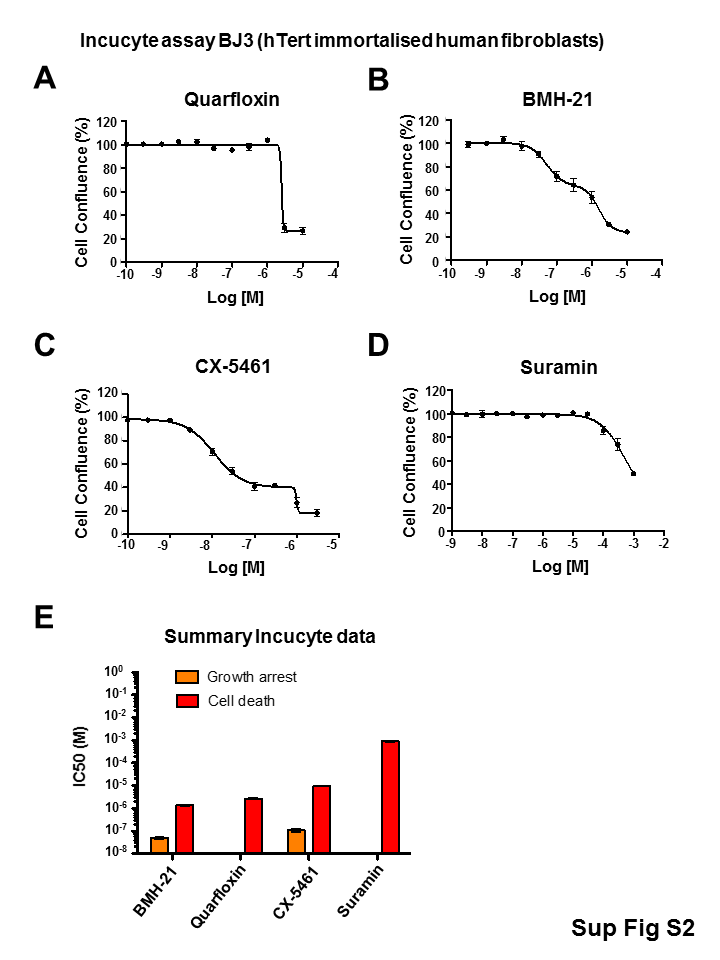

Supplement: S2 Fig — (A-D) Dose response curves of human foreskin fibroblasts immortalised by the addition of h-Tert and incubated for 48 hours with various doses of (A) quarfloxin, (B) BMH-21, (C) CX-5461 or (D) suramin. The mean percentage (%) of cell confluence is plotted from five biological replicates with the standard deviation indicated with error bars. (E) Summary of IC50 values for growth inhibition (where the higher doses prevent cell growth) and the cell death (where the higher doses cause cell death) (n = 5, error bars represent standard deviation). (TIF) [file pntd.0005432.s002.tif]

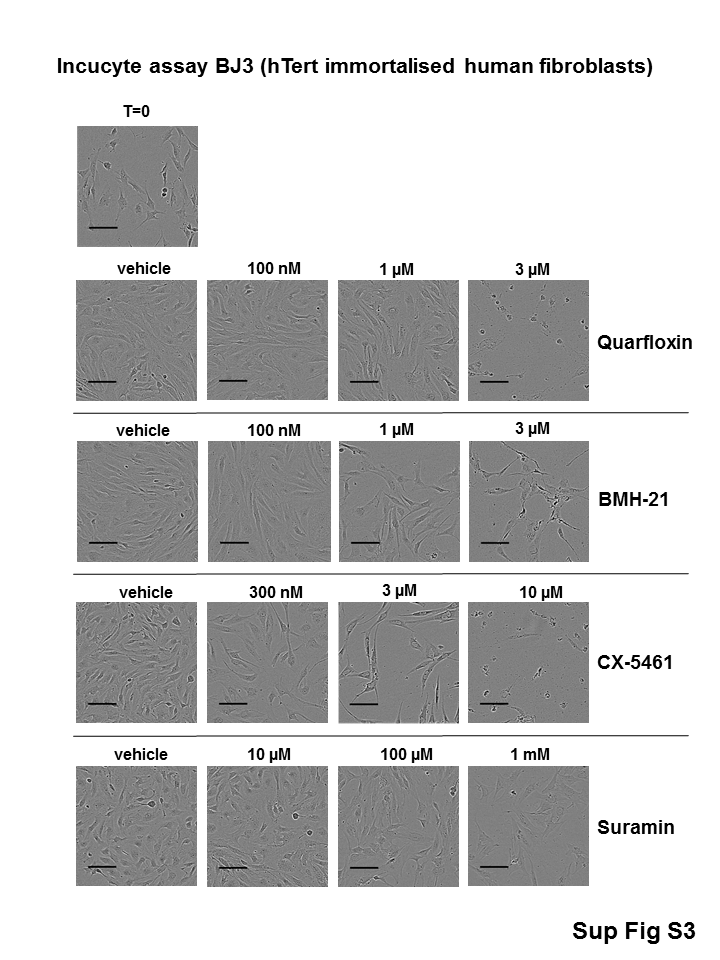

Supplement: S3 Fig — Cells were treated with various doses of quarfloxin, BMH-21, CX-5461 or suramin for 48 hours. Cells before treatment are shown (T = 0). Cells were imaged and cell confluence established using an IncuCyte ZOOM. Scale bars correspond to 100 μm. (TIF) [file pntd.0005432.s003.tif]

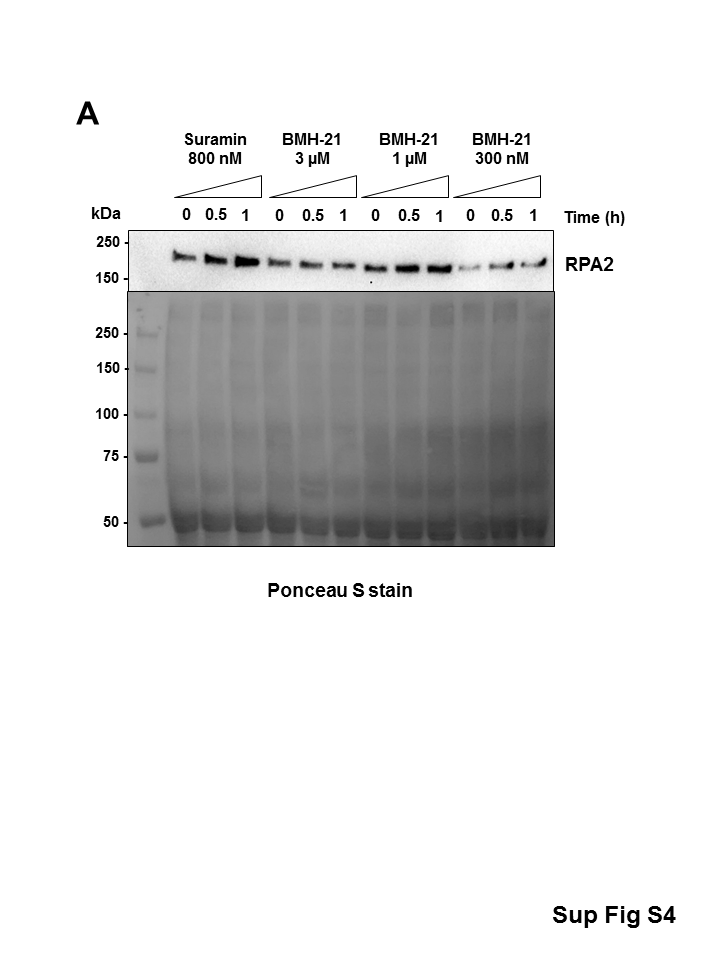

Supplement: S4 Fig — (A) The T. brucei TY-YFP-RPA2 cell line was incubated with various concentrations of BMH-21 for the time indicated in hours. As a control, T. brucei was also incubated with 800 nM of suramin. Lysate from 1 x 10^7^ cell equivalents was blotted and probed with anti-TY (BB2) antibody. A band corresponding to T. brucei RPA2 is shown, as well as a Ponceau S stain of the blot to serve as a loading control. Size markers in kiloDaltons (kDa) are indicated. (TIF) [file pntd.0005432.s004.tif]
