# Supplementary material for: Selective inhibition of RNA polymerase I transcription as a potential approach to treat African trypanosomiasis
Source: PLoS Negl Trop Dis. 2017 Mar 6;11(3):e0005432. doi: 10.1371/journal.pntd.0005432 (PMC5354456; doi:10.1371/journal.pntd.0005432)
Supplement: S1 Table — The gene location of the primers is shown, as well as the primer names and their DNA sequence (5’-3’). (PDF) [file pntd.0005432.s005.pdf]

Supplementary Table 1

List of primers used in qPCR experiments:

**Gene/Location**	**Primer name**	**Sequence (5'-3')**
rDNA primer a	18S_up_377s<br>18S_up_265as	CCATGCTCTCTCGTGTGTGTA<br>TTCCTCAAGGCGTCACTCTATC
rDNA primer b	28Sa_up_136s<br>28Sa_up_52as	AAAGAGGCGGCGGATAGTG<br>ACGAAAGAAGCACAAGCACATA
rDNA primer c	70srRNA_up_182s<br>70srRNA_up_68as	TTGAAGGGAATGCAAAAGTGTA<br>AACTGGAAGAGACGGAGGTAAA
rDNA primer d	140srRNA_up_89s<br>140srRNA_1as	TTGTGTTTCTATGTGTGTGTGTAAG<br>CGTTTGGAGAGGGACAAAATAT
VSG221 pre	Pre_221CTR_F2<br>Pre_221CTR_R2	TGGAGCGTACACACAAGTGA<br>ATGCATTGGCACACTTTCCG
VSG pseudo	Pseudo_221_f_kw<br>Pseudo_221_r_kw	GCAGGCAAGCATTACCAGAG<br>CTGTTCCGAATAGCGCGTC
Tubulin pre	αTub_up331s<br>αTub_up205as	GAGGAGGTGGGAAGGGTATATG<br>GAAGGCGTGTTGATGAGTTGTA
Actin	Actin_1031s<br>Actin_1091as	GTTCCATCCTCTCATCACTA<br>TCGTATTCACTCTTCGTTATC
